# Patent *Dirofilaria immitis* infection in Galapagos sea lion rookeries in San Cristóbal Island

**DOI:** 10.1017/S0031182025100425

**Published:** 2025-08

**Authors:** Carla A. Culda, Rommel Lenin Vinueza, Marjorie Riofrío-Lazo, Renato Leon, Diego Páez-Rosas, Andrei Daniel Mihalca

**Affiliations:** 1 Department of Parasitology and Parasitic Diseases, University of Agricultural Sciences and Veterinary Medicine of Cluj-Napoca, Cluj-Napoca, Romania; 2 Escuela de Medicina Veterinaria, Universidad San Francisco de Quito, Cumbayá, Ecuador; 3 Galapagos Science Center, Universidad San Francisco de Quito, Islas Galápagos, Ecuador; 4 Laboratorio de Entomología Médica & Medicina Tropical LEMMT, Universidad San Francisco de Quito, Cumbayá, Ecuador; 5 Fundación Conservando Galápagos, Galapagos Conservancy Inc, Islas Galápagos, Ecuador; 6 Dirección del Parque Nacional Galápagos, Unidad Técnica Operativa San Cristóbal, Islas Galápagos, Ecuador

**Keywords:** anthropic influences, canine heartworm, conservation medicine, Galapagos Islands, *Z. wollebaeki*

## Abstract

The Galapagos sea lion (*Zalophus wollebaeki*) is an endemic and endangered species that plays a vital role in the ecosystem dynamics of the archipelago. In recent decades, they have faced a significant population decline, related to the effects of climate variability and anthropogenic influences. Thus, the co-occurrence of sea lion resting areas with mosquito breeding sites and the presence of free-roaming domestic dogs present significant health risks related to parasite transmission. This research demonstrates the occurrence of *Dirofilaria immitis* (canine heartworm) in *Z. wollebaeki*, indicating their possible function as a definitive host for this parasite. Blood samples collected in August 2023 from 50 individuals (juveniles and adults) in 2 rookeries of San Cristóbal Island, revealed a 2% prevalence of *D. immitis* in juvenile females, as confirmed by Knott’s test and polymerase chain reaction analysis. Results of this work emphasize the critical necessity for effective monitoring and conservation strategies to address the threat posed by *D. immitis* and to safeguard this endangered species.

## Graphical abstract

**Figure:**
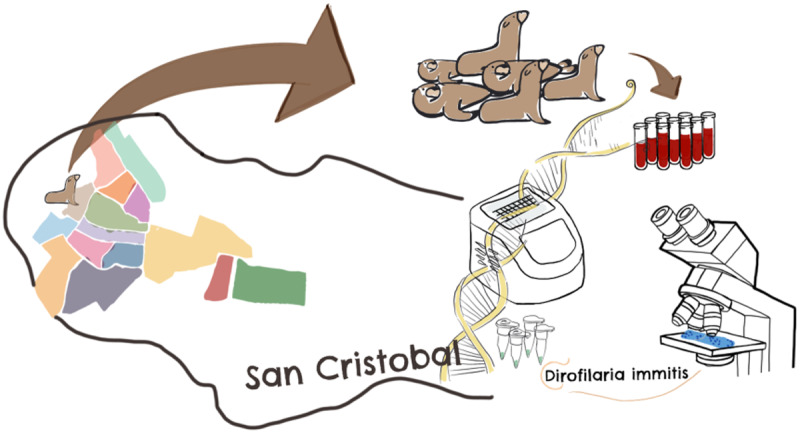

## Introduction

One of the most iconic species of the Galapagos Islands is the endemic and endangered Galapagos sea lion, *Zalophus wollebaeki* (Lorden et al., 2012; Trillmich, 2015). As the smallest sea lion species in the world, this otariid exhibits remarkable adaptations to the unique tropical environment of the archipelago (Trillmich et al., 2014; Riofrío-Lazo and Páez-Rosas, 2023). With a population of around 24 000 individuals (Páez-Rosas et al., 2021), this species has been evolutionarily separated from California sea lions, *Zalophus californianus*, for approximately 0.65 million years (Asadobay et al., 2023). The oceanographic dynamics of the archipelago were essential for the reproductive success and growth of its rookeries (Riofrío-Lazo and Páez-Rosas, 2021), such that the quality of its feeding areas is an indicator of environmental degradation, making it a sentinel of the ecosystem’s health in the region (Páez-Rosas and Guevara, 2017).

Their population has declined by about 50% over the past 4 decades, mainly due to climate variability effects, such as the El Niño-Southern Oscillation event (Kalberer et al., 2018; Páez-Rosas et al., 2021), along with anthropogenic influences (i.e. habitat degradation and introduced species) (Moreira-Mendieta et al., 2023; Ruiz-Saenz et al., 2023). The introduction of domestic animals, particularly dogs and cats, serves as an example of the impact of human activities on islands (Jimenez et al., 2020; Sarzosa et al., 2021). Free-roaming domestic dogs are present on all inhabited islands of Galapagos (i.e. San Cristóbal, Santa Cruz, Isabela and Floreana islands) (Culda et al., 2022; Diaz et al., 2016; Hernandez et al., 2020) and they represent important reservoirs for invasive pathogens that create new challenges for Galapagos sea lion populations (Culda et al., 2024; Jimenez et al., 2024; Vega-Mariño et al., 2023).

Interactions between Galapagos sea lions and domestic dogs pose health risks due to the potential transmission of pathogens (Denkinger et al., 2017; Sarzosa et al., 2021; Vega-Mariño et al., 2023; Walden et al., 2018). These include the canine distemper virus, parvoviruses, herpesviruses, caliciviruses, *Leptospira* or *Brucella* (Denkinger et al., 2017; Ruiz-Saenz et al., 2023). However, among the pathogens identified in dogs on the archipelago, *Dirofilaria immitis* has a potential impact on the pinniped populations of the region (Culda et al., 2022). The Galapagos sea lion rookeries on San Cristóbal Island are the largest population in the archipelago (Riofrío-Lazo et al., 2017). In El Malecón, a rookery close to the town of San Cristóbal Island, sea lions rest overlap with mosquito breeding sites, and dogs roam freely (Alagona, 2022; Culda et al., 2025), which increases the possibility of infections.

Barnett (1985) demonstrated the presence of microfilariae in the blood of Galapagos sea lions on Floreana, but without molecular confirmation and with no detailed on the larval morphology to allow clear identification as *D. immitis*. Recent studies have detected the presence of *D. immitis* in Galapagos sea lions through necropsy, antigen testing and DNA analysis (Gregory et al., 2023; Livingston et al., 2024). An action plan for eradicating the canine heartworm in the Galapagos was recently developed (Culda et al., internal report), which revolves around the question of whether domestic dogs represent the sole source of *D. immitis* infection for mosquitoes or Galapagos sea lions may also contribute to the spread of the parasite. In this context and with the recent findings of endemic foci of *D. immitis* on San Cristóbal Island (Gregory et al., 2023; Livingston et al., 2024), the current research aimed to evaluate the potential role of sea lions as hosts, which can develop a patent infection.

## Materials and methods

### Sample collection and examination

**Figure 1.**
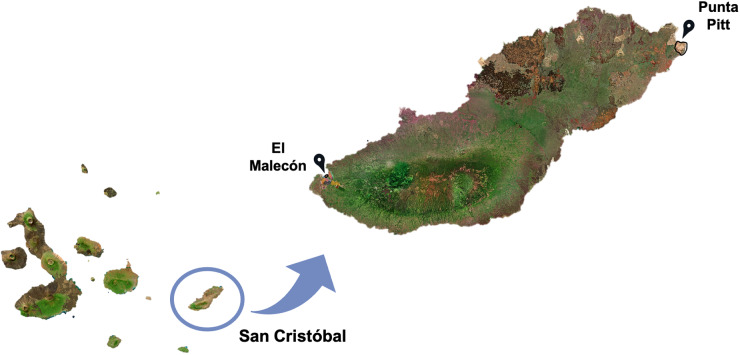
Sampling sites of Galapagos sea lions on San Cristóbal Island in August 2023.

The fieldwork and sample collection were carried out following the protocols of ethics and animal handling approved by the Galapagos National Park Directorate (GNPD) and the Universidad San Francisco de Quito (USFQ) under research permit PC-19-23. The sampling was conducted in August 2023 at 2 different rookeries on San Cristóbal Island. One site was located in the urban area of Puerto Baquerizo Moreno (El Malecón rookery, 0°54ʹ05.7″S and 89°36ʹ43.1″W, while the other site was in the protected natural area on the opposite side of the island (Punta Pitt rookery, −0°42ʹ59.4″S and 89°14ʹ47.2″W). A total of 50 blood samples were collected from both adult and juvenile Galapagos sea lions at these 2 rookeries (Figure 1; Supplementary File 1).

These 2 rookeries were selected to evaluate the level of exposure to pathogens and contact with domestic animals (dogs and cats). The distance between these 2 rookeries is approximately 50 km.

All animals captured in this study were monitored by a veterinarian and a GNPD ranger. The sea lions were weighed using an electronic scale, then removed from the net and restrained by experts handling this species. A physical examination was performed, and routine morphometric measurements were taken. Blood was collected from the caudal gluteal vein and aliquoted into citrate tubes. The samples were stored in a cooler and processed within 12 h. A total volume of 5 mL of blood was collected from each animal. Subsequently in the laboratory, the whole blood was divided into 2 equal aliquots: 0.5 mL in citrate tubes, for performing Knott’s test to evaluate the presence of *D. immitis* larvae (L1 – microfilariae) (Knott and Earle, 1939; Newton and Wright, 1956), the remaining 0.5 mL of blood was mixed with ethanol and kept at −20°C for further molecular analysis. The morphological differentiation of microfilariae was done according to Magnis et al. (2013) and Saari et al. (2019).

### DNA extraction and polymerase chain reaction amplification

Genomic DNA was extracted from blood samples using the DNeasy Blood & Tissue Kit (Qiagen, Germany), following the manufacturer’s protocol. A positive control was included in the DNA extraction process, which was sourced from the blood of a canine infected with *D. immitis*. The concentration and purity of the extracted DNA were assessed in duplicate using a NanoDrop ND-1000 spectrophotometer (NanoDrop Technologies, Inc., Wilmington, DE, USA).

Following the extraction, polymerase chain reaction (PCR) reactions were conducted to target different genes associated with various filarial species (Table 1). Each PCR reaction set included a positive control from an infected dog with *D. immitis* and a negative control that contained purified water instead of DNA. The positive samples were prepared to be sequenced by Macrogen Europe (Amsterdam, the Netherlands) and analysed using Geneious® 4.85 software and BLASTn to identify the closest matching sequences stored in GenBank®.

**Table 1.** Primer sequences used to identify filarial species and genes in Galapagos sea lions

Target gene	Primer	Sequence (5′-3′)	Amplicon size (bp)	Reference
*cox1* gene of filarial nematodes (including *D. immitis, D. repens* and *A. reconditum*)	DiCox	ACCGGTGTTTGGGATTGTTA	*D. immitis*, 170	Latrofa et al., 2012
	DrCox	GTATAATTTTGGGTTTACATACTGTA		
	ArCox	ATCTTTGTTTATGGTGTATC	*D. repens*, 480	
	NTR	ATAAGTACGAGTATCAATATC	*A. reconditum*, 590	
*5.8S-ITS2-28S* (including *D. immitis, A. reconditum, D. repens, A. dracunculoides, B. pahangi, B. malayi, B. timori, M. ozzardi* and *O. volvulus*)	DIDR-F1	AGTGCGAATTGCAGACGCATTGAG	*D. immitis*, 542	Rishniw et al., 2006
			*A. reconditum*, 578	
	DIDR-R1	AGCGGGTAATCACGACTGAGTTGA	*D. repens*, 484	
			*A. dracunculoides*, 584	
			*B. pahangi*, 664	
			*B. malayi*, 615	
			*B. timori*, 625	
			*M. ozzardi*, 430	
			*O. volvulus*, 470	

### Statistical analysis

The statistical analysis was performed using the EpiTools software. Age groups were established according to the potential risk of infection related to the duration of exposure to possible vectors for each sea lion. Odds ratios (ORs), 95% confidence intervals (CIs) and *P*-values were determined using univariate logistic regression to assess statistically significant prevalence differences. A *P*-value of ≤0.05 was considered statistically significant. Additionally, binomial proportions with 95% CIs were calculated for positive Galapagos sea lions.

## Results

**Figure 2.**
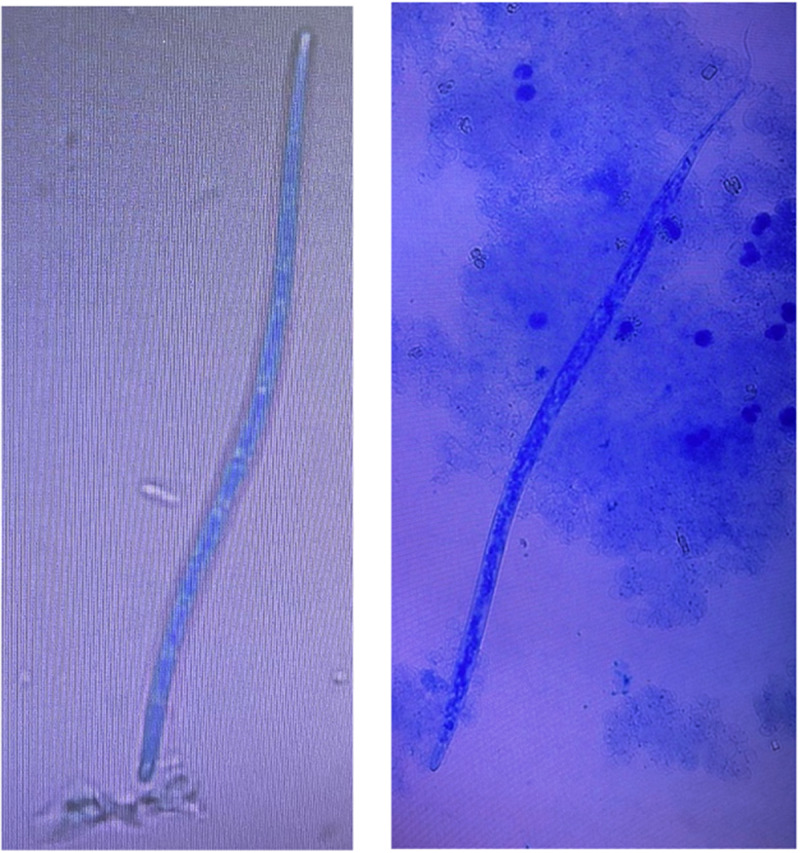
Microfilaria of *D. immitis* in circulating Galapagos sea lion blood from San Cristóbal Island.

From the 50 sea lion blood samples, one (prevalence 2.0%, 95% CI = 0–0.1) was microscopically positive for one microfilaria of *D. immitis*. The positive sample was collected from a juvenile female at the El Malecón rookery (Table 2; Figure 2). The anterior edge of circulating microfilariae was conical, with the nuclei situated at a distance from the cuticle. All blood samples were molecularly analysed using conventional PCR to identify possible filarial species, targeting 2 different genes: *cox1* and ITS2. The sample that tested positive in Knott’s test was successfully confirmed as *D. immitis* using PCR protocols. The resulting DNA sequences showed a similarity of 99–100% with available DNA sequences of *D. immitis* in the NCBI GenBank database (Table 3). All other samples were negative. The sequences obtained from the positive sample can be found in Supplementary File 2.

**Table 2.** Prevalence of microfilariae of *D. immitis* in Galapagos sea lion from San Cristóbal

Category		No. of sample	Positive (%)	OR	95% CI	*P-***value**
Sex	Males	11	0	NA	0 − 0.1	1
	Females	39	1 (2.6%)			
Age	Juvenil	19	1 (5.3%)	NA		0.9793
	Adult	20	0			
Rookery	El Malecón	25	1 (4%)	NA		1
	Punta Pitt	25	0			
Total		50	1 (2%)		0 − 0.1	

OR, odds ratio; 95% CI, 95% confidence interval; NA. not applicable.

**Table 3.** BLAST comparisons between the obtained sequences and the GenBank sequences (November 2024)

				GenBank entry					
Isolate	Organism	Gene	Sequence length (bp)	Query cover (%)	*E*-value	Percent identity* (%)	Host	Country	Similarity accession no
ZW50	*Dirofilaria immitis*	*cox1*	296	20	0.010	100	*Culex quinquefasciatus*	Portugal	MW246129
		ITS2	469	41	4E − 92	99.48	*Canis lupus familiaris*	Thailand	LC554219

* The percentage of identical nucleotides between the two sequences.

## Discussion

This study highlights new epidemiological data for *D. immitis* in Galapagos otariids. Worldwide, there are only few cases of heartworm infection reported in pinnipeds, all diagnosed by various methods from necropsy, antigen test, smear, modified Knott’s test to PCR and qPCR tests (Alho et al., 2017; Barnett, 1985; Farriols et al., 2020; Gregory et al., 2023; Jung et al., 2019; Kang et al., 2002; King, 1964; Livingston et al., 2024; Sato et al., 2002; White, 1975). Our findings, together with preliminary data by Barnett (1985), strongly suggest that the Galapagos sea lion can act as a suitable definitive host and reservoir for *D. immitis*. However, the duration of microfilariemia in sea lions is not known.

As shown in other non-canid hosts such as cats, the duration of microfilariemia and its intensity are significantly lower than in the preferred hosts, which are canids (American Heartworm Society, 2014; Simón et al., 2012). Recent findings revealed that *Culex quinquefasciatus* mosquitoes, known vectors for *D. immitis*, are feeding on Galapagos sea lions in the same area where circulating microfilariae were present in the sea lions’ blood (Culda et al., 2025). Additionally, *D. immitis* was identified by performing PCR tests on engorged mosquitoes near the Galapagos sea lion rookery (Culda et al., 2025). Another microfilariemic case was reported in pinnipeds, specifically in Cape fur seal, *Arctocephalus pusillus pusillus*, in an area highly endemic for canine dirofilariasis (Alho et al., 2017).

Our PCR successfully detected the presence of *D. immitis* in the blood of sea lions. Indeed, molecular analysis detected one sample as being positive for *D. immitis*, which was also positive for circulating microfilariae by Knott’s test. Recent studies have revealed that Galapagos sea lions’ blood has tested positive for *D. immitis* using antigen tests and PCR techniques (Gregory et al., 2023; Livingston et al., 2024). Both studies identified positive cases in the same rookeries of the current study, located at El Malecón. This study found no evidence in Punta Pitt, a remote area far from the port with minimal human interaction. Furthermore, Gregory et al. (2023) performed both morphological and molecular identification on 20 adult *D. immitis* worms recovered from the right ventricle of an adult Galapagos sea lion carcass found on Santa Cruz Island.

The El Malecón rookery where the positive sea lion was found is on one of the most populated islands in the archipelago, characterized by the presence of free-roaming dogs; ships and ferryboats; a high number of tourists attracted by local restaurants; and specific shops (Culda et al., 2022; Páez-Rosas and Guevara, 2017). Additionally, the presence of mangroves creates favourable conditions for mosquito breeding, all of which contribute to the transmission of *D. immitis* (Asigau and Parker, 2018; Asigau et al., 2019; Barnett, 1985; Culda et al., 2025). Previous studies in this island found a 1.7% prevalence of microfilariemia caused by *D. immitis* in dogs, based on a sample size of 587 animals (Culda et al., 2022).

The transmission cycle of *D. immitis* poses a significant threat to the endangered Galapagos sea lions. This was first noted in 1980 on Floreana Island, where *D. immitis* was detected in dogs, mosquitoes, Galapagos sea lions and even humans (Barnett, 1985). Floreana Island was the first to be colonized in the entire archipelago, allowing for the observation of how the delicate balance of island ecology can be disrupted by the changing life cycle of *D. immitis*. Gradually, other islands such as Isabela, San Cristóbal and Santa Cruz were also colonized, accompanied by the introduction of dogs and other invasive species. Currently, according to the International Union for Conservation of Nature, the settlements on San Cristóbal, Santa Cruz and Isabela Islands pose a significant risk of disease transmission from domestic carnivores to Galapagos fauna (Jimenez et al., 2024). This situation can be attributed to several factors, including the increase in the human population, the impact of tourism and administrative management by environmental authorities.

The evidence suggests that the Galapagos sea lion can act as a definitive host for *D. immitis*. Their high mobility raises the risk of the parasite spreading across the island, across various locations and potentially throughout the entire archipelago. Addressing these factors is crucial for creating a programme aimed at preventing this disease.

## Conclusion

This study reveals a new potential definitive host for *D. immitis* on San Cristóbal Island. Both the current study and previous research indicate that the dynamics of this multi-host parasite can pose a significant threat to Galapagos sea lions. Protecting this endemic and endangered species requires enhanced monitoring and conservation efforts. This knowledge is crucial for developing an effective eradication plan for canine heartworm and ensuring the long-term health of the region’s wildlife.

## Supporting information

Culda et al. supplementary material 1 Culda et al. supplementary material

Culda et al. supplementary material 2 Culda et al. supplementary material

## Data Availability

All data generated or analysed during this study are included in this publication.

## Supplementary material

The supplementary material for this paper can be found at https://doi.org/10.1017/S0031182025100425.
