# Supplementary material for: Patent Dirofilaria immitis infection in Galapagos sea lion rookeries in San Cristóbal Island
Source: Parasitology. 2025 Jul 10;152(9):932–7. doi: 10.1017/S0031182025100425 (PMC12644960; doi:10.1017/S0031182025100425)
Supplement: Culda et al. supplementary material 1 — Culda et al. supplementary material [file S0031182025100425sup001.docx]

Galapagos sea lions captured on San Cristóbal Island rookeries

| **Rookery** | **Age class** | **Sex** | **Weight (kg)** |
| --- | --- | --- | --- |
| Punta Pitt | Adult | Female | na^*^ |
|  | Adult | Female | na^*^ |
|  | Adult | Female | na^*^ |
|  | Adult | Female | na^*^ |
|  | Adult | Female | na^*^ |
|  | Adult | Female | na^*^ |
|  | Adult | Female | na^*^ |
|  | Adult | Female | na^*^ |
|  | Adult | Female | na^*^ |
|  | Adult | Female | na^*^ |
|  | Juvenile | Female | 30.1 |
|  | Juvenile | Female | 28.6 |
|  | Juvenile | Male | 22.3 |
|  | Juvenile | Female | 48.3 |
|  | Juvenile | Male | 48.3 |
|  | Juvenile | Female | 53.1 |
|  | Juvenile | Female | 44.8 |
|  | Juvenile | Female | 35.7 |
|  | Juvenile | Male | 60 |
|  | Juvenile | Female | 25.2 |
|  | Juvenile | Male | 49 |
|  | Juvenile | Female | 41.3 |
|  | Juvenile | Female | 33 |
|  | Juvenile | Male | 22.0 |
|  | Juvenile | Female | 48.3 |
| El Malecón | Adult | Female | na^*^ |
|  | Adult | Female | na^*^ |
|  | Adult | Female | na^*^ |
|  | Adult | Female | 54.1 |
|  | Adult | Female | 46.3 |
|  | Adult | Female | na^*^ |
|  | Adult | Female | na^*^ |
|  | Adult | Female | na^*^ |
|  | Adult | Female | na^*^ |
|  | Adult | Female | na^*^ |
|  | Juvenile | Female | 22.7 |
|  | Juvenile | Male | 32.7 |
|  | Juvenile | Female | 28.9 |
|  | Juvenile | Male | na^*^ |
|  | Juvenile | Male | 30.9 |
|  | Juvenile | Female | 44.4 |
|  | Juvenile | Male | 14.0 |
|  | Juvenile | Male | 39.8 |
|  | Juvenile | Male | 31.5 |
|  | Juvenile | Female | 38.1 |
|  | Juvenile | Female | 35.6 |
|  | Juvenile | Female | 26.0 |
|  | Juvenile | Female | 22.9 |
|  | Juvenile | Female | 26 |
|  | Juvenile | Female | 20.2 |

*na= not available
